# Towards a better understanding of the cellular basis for cerebrospinal fluid shunt obstruction: report on construction of a bank of explanted hydrocephalus devices

**DOI:** 10.1186/2045-8118-12-S1-O17

**Published:** 2015-09-18

**Authors:** Brian W Hanak, Emily F Ross, Carolyn A Harris, Samuel R Browd, William Shain

**Affiliations:** 1 University of Washington, Seattle, WA, USA; 2 Wayne State University, Detroit, MI, USA

## Introduction

Shunt obstruction with cells/tissue is the most common cause of shunt failure; with ventricular catheter obstruction, alone, accounting for >50% of pediatric failures. We sought to systematically collect explanted ventricular catheters from Seattle Children's Hospital with a focus on elucidating cellular mechanisms underlying obstruction.

## Methods

In the operating room explanted hardware was placed in 4% paraformaldehyde. Weekly, samples were transferred to buffer solution and stored at 4°C. After obtaining consent/assent, catheters were labeled using cell-specific markers for astrocytes (monoclonal rat anti-glial fibrillary acidic protein), microglia (monoclonal rabbit anti-Iba1), and choroid plexus (polyclonal chicken anti-transthyretin) for 24 hrs. These targets were visualized using goat anti-rat Alexa 488, goat anti-rabbit Alexa 594, and goat anti-chicken Alexa 647 conjugated secondary antibodies, which were applied for 24 hrs in conjunction with a nuclear stain (Hoechst). Catheters were mounted in custom polycarbonate imaging chambers. Three-dimensional, multispectral spinning disk confocal microscopy was utilized to image catheters (Olympus, IX81 inverted microscope, motorized stage, charged-coupled camera).

## Results

Intraoperatively confirmed ventricular catheter obstruction was the leading cause of shunt failure, noted in 53.6% of cases. Shunt hardware was explanted in 321 surgeries during the study period (4/1/13 – 11/30/14) and we received hardware in 34.0% of cases. Our consent rate for explanted ventricular catheters was 58.2%. Bugbee wire monopolar electrocautery was used on 26.1% of explanted catheters. Over 30 ventricular catheters have been imaged to date, resulting in the following observations: 1) Astrocytes and microglia are the dominant cell types bound directly to catheter surfaces; 2) Cellular binding to catheters is ubiquitous even if no grossly visible tissue is apparent; 3) Commercially available catheters contain rough, irregular surfaces, particularly at CSF intake holes, and there appears to be preferential cell binding to these rough surfaces; 4) Immunohistochemistry techniques are of limited utility when a catheter has been exposed to Bugbee wire electrocautery.

## Conclusions

Ventricular catheter occlusion remains a significant source of shunt morbidity in the pediatric population and, given their ability to intimately associate with catheter surfaces, astrocytes and microglia appear to be critical to this pathophysiology. Reduced shunt failure rates may be possible through improved ventricular catheter design. Work is ongoing to fabricate catheters with smooth CSF intake portals and altered surface chemistry, with the goal of making catheters a less favorable substrate for cell attachment.
